# Regional gene expression and brain atrophy in dementia with Lewy bodies: an imaging transcriptomics study

**DOI:** 10.1038/s41531-026-01355-2

**Published:** 2026-04-16

**Authors:** Annegret Habich, Janna M. Baumann, Christopher G. Schwarz, Scott A. Przybelski, Anna Inguanzo, Ketil Oppedal, Frédéric Blanc, Afina W. Lemstra, Jakub Hort, Bradley F. Boeve, Dag Aarsland, Eric Westman, Thomas Dierks, Kejal Kantarci, Laura E. Jonkman, Daniel Ferreira

**Affiliations:** 1 Division of Clinical Geriatrics, Center for Alzheimer Research, Department of Neurobiology, Care Sciences and Society, Karolinska Institute, Stockholm, Sweden; 2 University Hospital of Psychiatry and Psychotherapy Bern, University of Bern, Bern, Switzerland; 3 Vrije Universiteit Amsterdam, Amsterdam, Netherlands; 4 Department of Radiology, Mayo Clinic, Rochester, MN USA; 5 Quantitative Health Sciences, Mayo Clinic, Rochester, MN USA; 6 Department of Electrical Engineering and Computer Science, University of Stavanger, Stavanger, Norway; 7 Day Hospital of Geriatrics, Memory Resource and Research Centre (CM2R) of Strasbourg, Department of Geriatrics, Hopitaux Universitaires de Strasbourg, Strasbourg, France; 8 ICube Laboratory and Federation de Medecine Translationnelle de Strasbourg (FMTS), University of Strasbourg and French National Centre for Scientific Research (CNRS), Team Imagerie Multimodale Integrative en Sante (IMIS)/ICONE, Strasbourg, France; 9 Amsterdam Alzheimer Center, Department of Neurology, Amsterdam University Medical Centers, Vrije Universiteit, Amsterdam, Netherlands; 10 Memory Clinic, Department of Neurology, Charles University, Second Faculty of Medicine and Motol University Hospital, Prague, Czech Republic; 11 Department of Neurology, Mayo Clinic, Rochester, MN USA; 12 Department of Old Age Psychiatry, Institute of Psychiatry, Psychology & Neuroscience, King’s College London, London, UK; 13 Center for Age-Related Medicine, Stavanger University Hospital, Stavanger, Norway; 14 Department of Anatomy and Neurosciences, Section Clinical Neuroanatomy and Biobanking, Amsterdam UMC, Amsterdam, Netherlands; 15 Amsterdam Neuroscience, Brain Imaging, Amsterdam, Netherlands; 16 Department of Psychology, Faculty of Health Sciences, University Fernando Pessoa Canarias, Las Palmas, Spain

**Keywords:** Biomarkers, Diseases, Neurology, Neuroscience

## Abstract

Regional brain atrophy has been observed in dementia with Lewy bodies (DLB), yet determinants of regional vulnerability remain unclear. Using imaging transcriptomics, we examined whether normative gene expression patterns relate to regional atrophy in DLB. We included 164 DLB patients (49 women) and 164 age- and sex-matched healthy controls from three European centres and the Mayo Clinic, USA. Volumetric atrophy was quantified from T1-weighted MRI across 58 left-hemispheric regions using *w*-scores. Normative expression of twelve genes implicated in alpha-synuclein, beta-amyloid, and tau pathology was extracted from the Allen Human Brain Atlas. DLB patients showed diffuse atrophy across most regions. In the full cohort, normative expression of *MAPT*, *PINK1*, and *PSEN2* predicted regional atrophy after correction for spatial autocorrelation, although none survived multiple-testing correction. In the Mayo Clinic sub-cohort, expression of *APP*, *BIN1*, *GBA*, *MAPT*, *PINK1*, *SNCA*, and *TMEM175* significantly predicted atrophy and survived multiple-testing correction. Random forest models did not outperform spatial null models in the full cohort, but *PARK7*, *PINK1*, and *PSEN2* consistently emerged as important predictors. A significant global model was observed in the Mayo Clinic sub-cohort, driven by *GBA*, *LRP1*, and *PINK1*. These findings suggest that normative gene expression partially contributes to regional brain atrophy in DLB.

## Introduction

In dementia with Lewy bodies (DLB), atrophy is typically less pronounced than in Alzheimer’s disease (AD). Nevertheless, patients affected by DLB have been shown to exhibit grey matter reductions in frontal, temporal, parietal, and occipital lobes as well as in subcortical structures compared with healthy controls^1–3^. These observed reductions in regional grey matter volumes and cortical thickness in magnetic resonance imaging (MRI) have been associated with the presence and severity of several core clinical features in DLB, i.e., REM-sleep behaviour disorder (RBD), parkinsonism, visual hallucinations, and cognitive fluctuations^4–7^.

Animal studies suggest that the observed neurodegenerative processes are linked to the formation and accumulation of clusters of misfolded α-synuclein, forming the neurotoxic Lewy bodies^8–10^. According to post-mortem Braak staging, the α-synuclein pathology first accumulates in the brainstem and limbic regions before spreading to cortical structures^11,12^. Although many brain regions that accumulate α-synuclein pathology show atrophy, the regional overlap between histopathological findings and imaging markers of atrophy seems to be incomplete. Partially, this incongruence may be driven by the common beta-amyloid and p-tau (A/T) co-pathologies observed in up to 60% of DLB patients and the associated neurodegenerative processes^13,14^. In fact, concomitant A/T pathologies were associated with a more pronounced medial temporal lobe and posterior atrophy in DLB patients compared with patients without these co-pathologies^15,16^.

Previous studies in other alpha-synucleinopathies, specifically idiopathic REM-sleep behaviour disorder and Parkinson’s disease (PD), demonstrated that genes associated with the synthesis, transfer, and degradation of alpha-synuclein predicted regional brain atrophy^17–21^. Genome-wide association studies revealed that many of the genes that determine the vulnerability of brain regions to atrophy coincide with gene risk loci for DLB^22–25^. As expected due to their direct involvement in the production and degradation of alpha-synuclein, *SNCA* and *GBA* were repeatedly indicated as important determinants in the neurodegenerative processes in DLB. Similarly, *PARK7* and *PINK1* are involved in the removal of alpha-synuclein^26,27^. Moreover, several studies emphasized that disruptions in lysosomal activity determined by the translational products of *BIN1* and *TMEM175* genes are linked to DLB-related alpha-synuclein pathogenesis^28,29^. In line with the common A/T co-pathologies, genes such as *APOE*, *APP*, *LRP1*, *MAPT*, *PSEN1* and *PSEN2* that are related to the production and accumulation of beta-amyloid and the formation and propagation of tau fibrils may also exert an influence on atrophy patterns in DLB^30–33^.

As genes are differentially expressed across brain regions, these findings imply a region-specific vulnerability to the alpha-synuclein pathology, co-pathologies and their respective neurotoxic effects in DLB. However, the extent to which normative regional gene expression profiles contribute to vulnerability to atrophy in DLB remains largely unexplored. To close this gap, we used an imaging transcriptomics approach. First, we aimed to assess the individual association of the normative expression of each of the twelve genes with regional grey matter volumes in DLB. Secondly, considering the complex relationship between the regional expression of different genes, we assessed the combined capacity of the twelve genes to predict regional grey matter volume in DLB. We hypothesised that brain regions with specific normative transcriptomic profiles related to alpha-synuclein and A/T co-pathologies would exhibit increased vulnerability to regional atrophy in DLB.

## Results

### Cohort characteristics

In our cohort, the 164 DLB patients and 164 cognitively unimpaired healthy controls were matched in age and sex (Tab. 1). DLB patients received a shorter education and scored lower on the Mini-Mental State Examination (MMSE). Clinical core features, including probable RBD (77.9%), parkinsonism (87.0%), visual hallucinations (55.3%), and cognitive fluctuations (83.3%), were highly prevalent among the DLB patients. Additionally, more than half (54.1%) of the DLB patients were positive for beta-amyloid and/or tau. Characteristics of the subgroups are described in Table 1.

**Table 1:** Demographic and clinical characteristics of DLB patients and healthy controls

Variables	DLB patients (*n* = 164)	DLB patients ^A/T-^(*n* = 56)	DLB patients ^A/T+^ (*n* = 66)	DLB patients ^Mayo^ (*n* = 67)	Healthy controls (*n* = 164)	Statistics
Age (years)	69.1 ± 8.6	67.2 ± 8.9	69.7 ± 8.3	69.5 ± 8.0	69.0 ± 8.6	p = 1.00 (*t*-test)
Women/men (% women)	45/119 (27.4%)	21/35 (37.5%)	16/50 (24.2%)	10/57 (14.9%)	45/119 (27.4%)	*p* = 1.00 (χ^2^-test)
Education (years)	13.3 ± 3.9	13.0 ± 4.2	13.3 ± 3.8	15.4 ± 3.1	15.1 ± 2.7	***p*** < **0.001** (*t*-test)
MMSE	22.9 ± 5.2	24.8 ± 4.0	22.9 ± 4.4	22.0 ± 6.1	28.5 ± 1.2	***p*** < **0.001** (*t*-test)
TIV	1579.3 ± 156.0	1541.0 ± 157.2	1603.4 ± 157.7	1606.6 ± 136.2	1551.2 ± 155.2	*p* = 0.103 (*t*-test)
Probable RBD	116 (77.9%)^a^	40 (80.0%)^f^	39 (68.4%)^g^	60 (92.3%)^b^	n.a.	
Parkinsonism	141 (87.0%)^b^	48 (85.7%)	53 (82.8%)^b^	61 (93.8%)^b^	n.a.	
Visual hallucinations	89 (55.3%)^c^	28 (50.0%)	30 (47.6%)^c^	42 (64.6%)^b^	n.a.	
Cognitive fluctuations	130 (83.3%)^d^	46 (85.2%)^b^	49 (81.7%)^f^	55 (84.6%)^b^	n.a.	
A/T co-pathologies	66 (54.1%)^e^	0 (0%)	66 (100%)	15 (55.6%e)^j^	n.a.	

For continuous variables, data is provided as mean ± standard deviation. For categorical variables, count and percentage is provided. Statistics pertain to comparison between all DLB patients (*n* = 164) and healthy controls. Missing data for ^a^*n* = 15, ^b^*n* = 2, ^c^*n* = 3, ^d^*n* = 8, ^e^*n* = 42, ^f^*n* = 6, ^g^*n* = 9, ^h^*n* = 4, ^i^*n* = 1, ^j^*n* = 40 DLB patients. *AD* Alzheimer’s disease, *HC* healthy controls, *MMSE* Mini Mental State Examination, *n.a.* not available. *RB*D = REM sleep behaviour disorder. *TIV* total intracranial volume.

### Regional grey matter volumetric scores

**Fig. 1:**
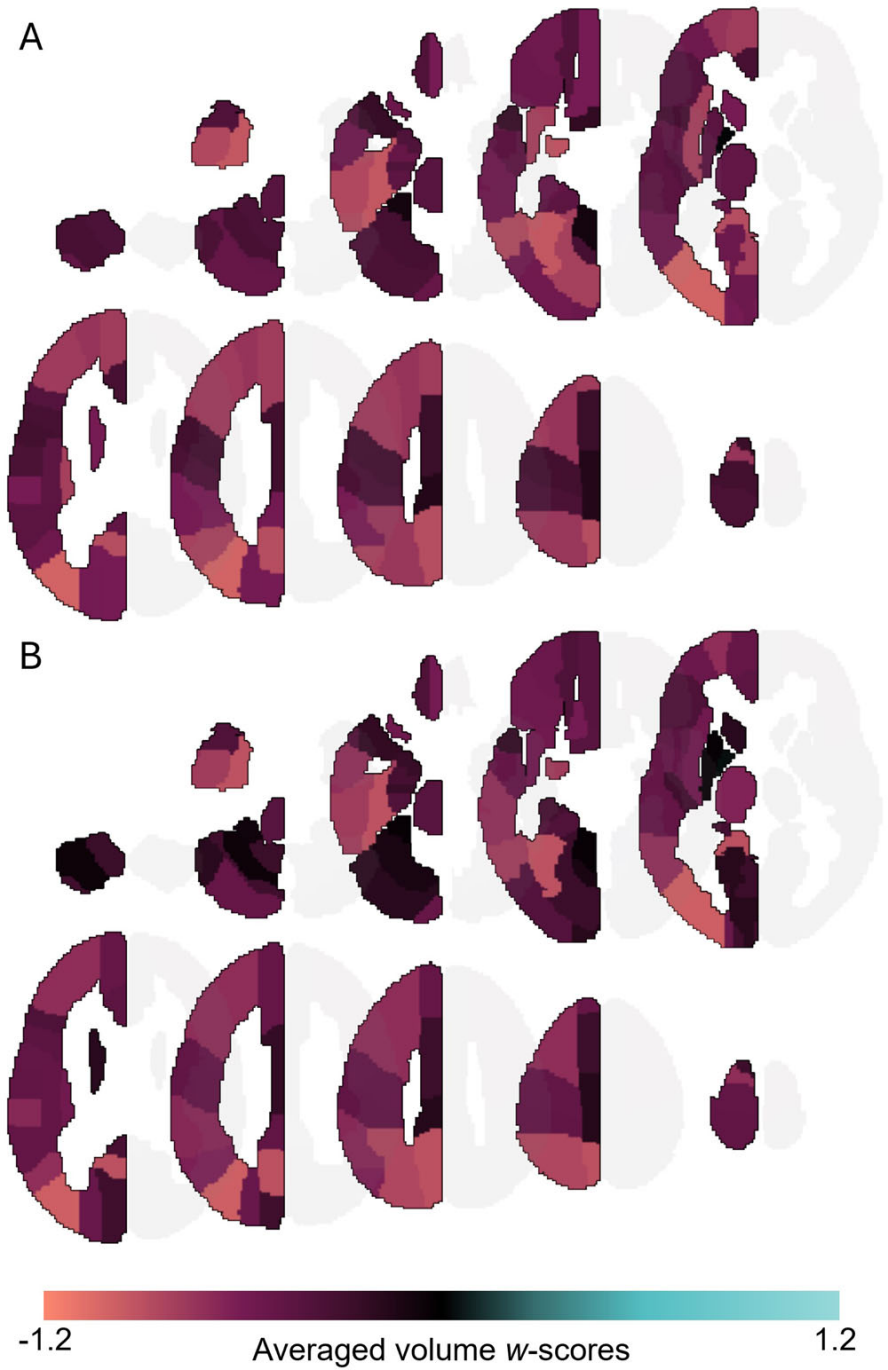
Atrophy pattern in DLB. Average regional volume *w*-scores in left hemisphere of **A** all DLB patients (*n* = 164) and **B** DLB patients from the Mayo Clinic (*n* = 67) compared to healthy controls.

T-tests on regional volume w-scores across all 164 DLB patients indicated a diffuse pattern of atrophy compared to healthy controls (Fig. 1, Table S2). Atrophy affected all regions in frontal, temporal, parietal, and occipital lobes, as well as most subcortical, cerebellar, and brainstem regions. Similar patterns of reduced regional volumes emerged in subgroups of DLB patients from the Mayo Clinic, as well as subgroups of DLB patients with and without A/T co-pathologies (Fig. S1, Table S2).

### Normative regional gene expression

**Fig. 2:**
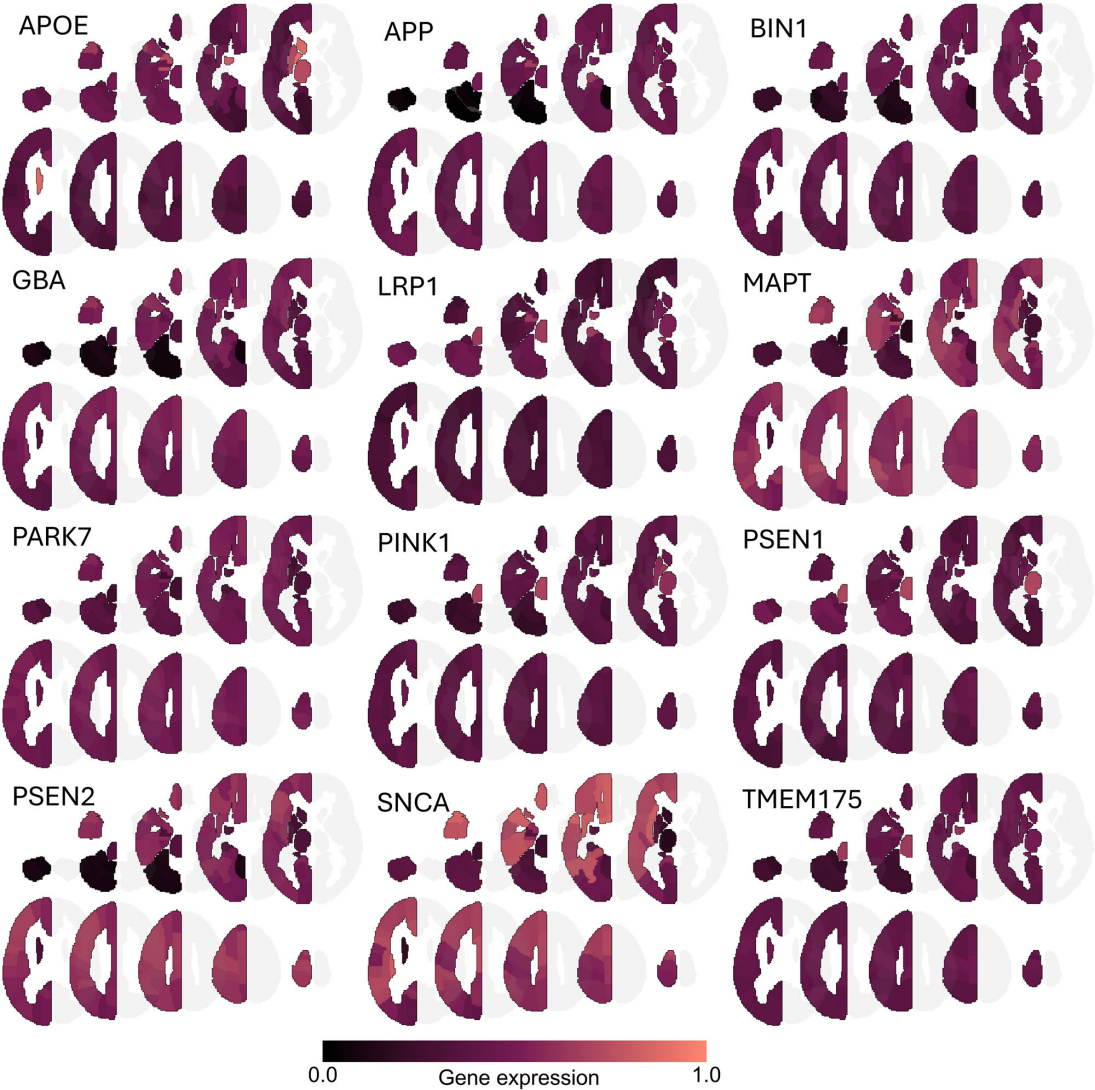
Normative gene expression pattern of twelve included genes in left hemisphere. Data was extracted from the Allen Human Brain Atlas.

The twelve selected genes differed in normative regional expression patterns (Fig. 2, Table S3). Visual inspection revealed that *APOE* was most highly expressed in subcortical structures and moderately expressed in cortical, cerebellar, and brainstem regions. *APP, BIN1, and GBA* showed uniform expression patterns in cortical, subcortical, and brainstem regions, with lower expression values in cerebellar regions. *LRP1*, *PINK1*, *PSEN1*, and *TMEM175* were most highly expressed in subcortical regions and brainstem and more moderately in all other regions. *MAPT*, *PARK7*, *PSEN2*, and *SNCA* showed higher expression levels in cortical regions, whereas subcortical and cerebellar regions exhibited lower expression values. Expression of the twelve genes significantly correlated across brain regions (Fig. S2).

### Association of normative gene expression with regional grey matter volumes in DLB

**Fig. 3:**
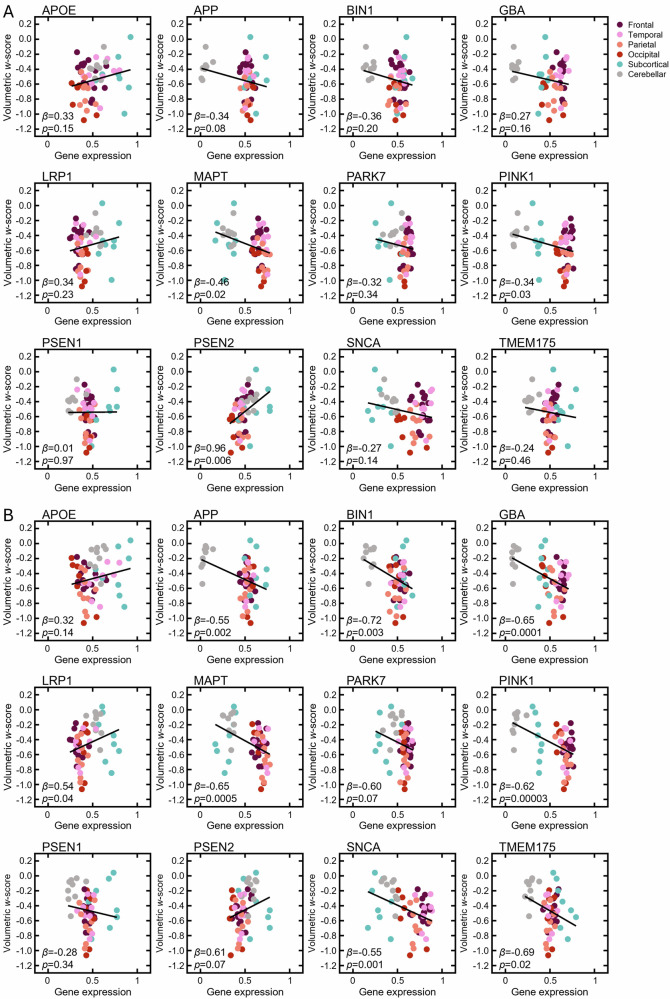
Association between normative expression of individual genes and atrophy (w-scores). Simple linear regression across 58 brain regions in **A** all DLB patients (*n* = 164) and **B** DLB patients from the Mayo Clinic (*n* = 67). Colours denote regions’ affiliation with lobes and major brain structures. Panels include beta coefficients *β* and *p*-values adjusted for spatial autocorrelation *p*_spatial_.

To address aim 1 of our study, we assessed the association of the normative expression of the twelve genes with regional grey matter volume in DLB, individually. For the entire DLB cohort, higher regional normative gene expression of *MAPT*, *PINK1*, and *PSEN2* significantly predicted grey matter volume *w*-scores after correction for spatial autocorrelation. None of the three genes survived Benjamin-Hochberg correction (Fig. 3A). A similar pattern was found in DLB patients without and with A/T co-pathologies (Fig. S3A, B). Using only data from the Mayo Clinic DLB cohort to preclude centre effects, normative regional expression of *APP* (*β* = −0.55, SE = 0.17, *t*_(56)_ = −3.23, *p*_spatial_ = 0.008, *R*^2^ = 0.16), *BIN1*(*β* = −0.72, SE = 0.24, *t*_(56)_ = −3.01, *p*_spatial_ = 0.02, *R*^2^ = 0.14), *GBA* (*β* = −0.65, SE = 0.16, *t*_(56)_ = −4.03, *p*_spatial_ = 0.002, *R*^2^ = 0.22), *MAPT* (*β* = −0.65, SE = 0.18, *t*_(56)_ = −3.70, *p*_spatial_ = 0.003, *R*^2^ = 0.20), *PINK1* (*β* = −0.62, SE = 0.14, *t*_(56)_ = −4.51, *p*_spatial_ = 0.0001, *R*^2^ = 0.27), *SNCA* (*β* = −0.55, SE = 0.16, *t*_(56)_ = −3.39, *p*_spatial_ = 0.001, *R*^2^ = 0.17), and *TMEM175* (*β* = −0.69, SE = 0.29, *t*_(56)_ = −2.40, *p*_spatial_ = 0.02, *R*^2^ = 0.09) significantly predicted grey matter volume *w*-scores that all survived correction for multiple testing (Fig. 3B).

**Fig. 4:**
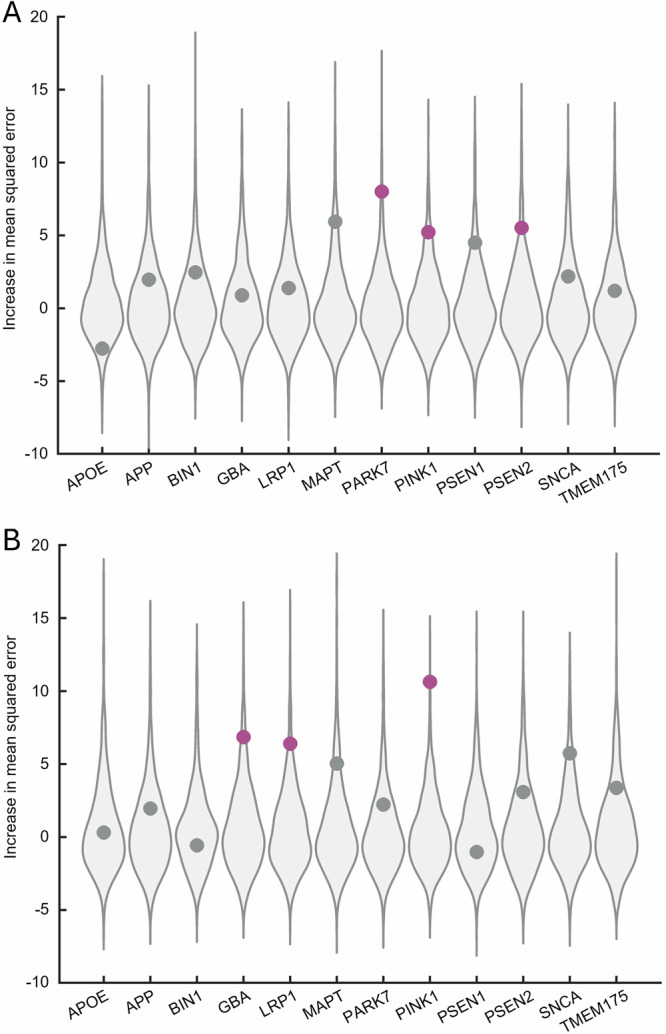
Association between normative expression of twelve genes and atrophy (w-scores). Random forest regression analysis across 58 brain regions in **A** all DLB patients (*n* = 164) and **B** DLB patients from the Mayo Clinic (*n* = 67). Circular markers represent empirical importance of each gene with plum markers indicating significance *p*_spatial_ < 0.05 relative to null distribution. Grey violins represent null distributions of variable importance across 1’000 surrogate maps.

To address aim 2 of our study, we assessed the combined prediction of normative expression of the twelve genes on regional grey matter volume in DLB using random forest regression. Including the average regional volumes from the entire DLB cohort, the global random forest model did not exceed the null threshold (*R*^2^ = 0.096, OOB MSE = 0.052, Var(y)=8.06%, *p*_spatial_ = 0.07). However, a subset of genes, specifically *PARK7* (*p*_spatial_ = 0.014), *PINK1* (*p*_spatial_ = 0.04), and *PSEN2* (*p*_spatial_ = 0.05), outperformed their respective spatial null distributions (Fig. 4). Similarly, the global random forest model performance did not reach significance in DLB patients without (*R*^2^ = 0.092, OOB MSE = 0.054, Var(y)= 7.620%, *p*_spatial_ = 0.12; Fig. S4A) and with (*R*^2^ = 0.064, OOB MSE = 0.08, Var(y)= 4.7%, *p*_spatial_ = 0.11; Fig. S4B) A/T co-pathologies. Despite the global models not being significant, a qualitative inspection showed that the most important predictor in both subgroups was *PARK7*. The global model was, however, significant in the subgroup-cohort of DLB patients from the Mayo Clinic (*R*^2^ = 0.268, OOB MSE = 0.05, Var(y)=25.53%, *p*_spatial_ = 0.004; Fig. S4C) with *GBA* (*p*_spatial_ = 0.04), *LRP1* (*p*_spatial_ = 0.03), and *PINK1* (*p*_spatial_ = 0.004) being the best predictors.

## Discussion

In this study, we followed an imaging transcriptomics approach to investigate whether the regional vulnerability to atrophy in DLB is associated with the normative regional expression profiles of twelve genes. While previous studies in related synucleinopathies, i.e., idiopathic REM sleep behaviour disorder and PD, demonstrated that normative regional gene expression may underlie the susceptibility to regional atrophy in such diseases^19,20^, this is the first study to demonstrate a similar relationship in DLB. In contrast to previous studies, we not only included genes related to the synthesis and degradation of alpha-synuclein but also included genes related to beta-amyloid and tau in our analyses.

For our first aim, we conducted linear regression analyses to investigate whether differential normative expression of 12 candidate genes relates to the observed pattern of atrophy in DLB patients. When considering all DLB patients, we observed only trending associations between regional normative expression of *MAPT*, *PINK1*, and *PSEN2* and regional atrophy in DLB patients. In contrast, the analysis of the more homogenous sub-cohort of patients from the Mayo Clinic revealed robust associations between normative regional expressions of various genes related to alpha-synuclein (*GBA*, *PINK1*, *SNCA*, *TMEM175*) and regional atrophy. While *SNCA* is involved in the synthesis of alpha-synuclein, expression products of *GBA* and *PINK1* contribute to its degradation. Similarly, *TMEM175* deficiency was previously shown to increase the pathological aggregation of alpha-synuclein^28^. While alpha-synuclein is considered the characteristic pathology underlying DLB, with variants of *SNCA* playing a pivotal role in the early stages of alpha-synuclein pathology^34^, the co-existence of beta-amyloid and tau pathologies has been shown in both in-vivo and post-mortem samples of DLB patients^14,35^. Importantly, the presence of both pathologies in DLB patients has been suggested to lead to an accelerated cognitive decline while also shaping the rate and pattern of brain atrophy^16,36,37^.

In accordance with these previous findings, the normative overexpression of three genes related to tau (*APP*, *BIN1*, *MAPT*) predicted vulnerability to atrophy in DLB patients from the Mayo Clinic. Both *APP* and *BIN1* were previously shown to play crucial roles in tau propagation^38,39^. Similarly, *MAPT* mutations may increase the probability of pathological tau aggregation^31^. Importantly, AD-related pathologies do not merely co-exist with alpha-synuclein and drive disease progression independently. Instead, beta-amyloid and tau monomers were shown to facilitate the aggregation of alpha-synuclein^30,40–42^. Our findings indicate that the atrophy pattern in DLB emerges from the convergence of pathological pathways involving multiple pathologies.

For our second aim, we conducted random forest regression analyses to assess the combined predictive power of the normative expression of twelve genes on grey matter volume *w*-scores across brain regions in DLB. When analysing data of all DLB patients, the global random forest model did not exceed the spatial null threshold. However, even in the absence of a significant global fit, normative regional expression of *PARK7*, *PINK1*, and *PSEN2* outperformed their spatial null distributions. Proteins encoded by normal variants of *PARK7* and *PINK1* are involved in the removal of alpha-synuclein, whereas proteins expressed by mutant variants of the genes may increase the vulnerability to the alpha-synuclein pathology^26,27^. In contrast, mutated variants of *PSEN2* are related to the accumulation of amyloid pathology^43,44^. Of note, gene expression that was most important to predict regional volume reductions was not exclusively related to alpha-synuclein accumulation but also amyloid accumulation. Previous studies have shown that DLB and its prodromal stages share a combination of genetic risk loci of AD and PD, primarily implicating *APOE*, *GBA*, and *SNCA*^22,25,45–48^. While the regional expressions of these genes were not included as the most significant predictors for grey matter volumes when considering all included DLB patients, our findings nevertheless point to the involvement of genes related to both AD and PD. The biological validity of this result was confirmed in the Mayo Clinic sub-cohort, where the global random forest regression model was significant. In this more homogeneous sample, the normative regional expression of the 12 genes explained over 25% of the spatial variance in atrophy observed in DLB patients. Normative regional expression of three genes (*GBA*, *LRP1*, *PINK1*) outperformed their spatial null distributions, with *PINK1* being the most important predictor. These results included the previously indicated risk genes *GBA*, as well as other genes related to the accumulation of alpha-synuclein, beta-amyloid, and tau (*LRP1*, *PINK1*)^24,27,49^. Notwithstanding that, except for *PINK1* the most important contributors to the random forest model did not match between the full cohort and the Mayo Clinic sub-cohort; the combined results reinforce the notion of complex interactions between various pathologies in DLB.

Comparing the results of our linear and random forest regression analyses reveals a partial overlap in genetic contributors, most notably with *PINK1* emerging as a consistent factor in both models. However, the divergence in results, with certain genes contributing only to the random forest model, suggests a more complex relationship. This discrepancy suggests that while normative expression of some genes may not show significant independent correlations with grey matter volume (i.e., linear regression tests for partial effects), their synergistic normative expression seems to contribute to regional vulnerability to atrophy (i.e., random forest accommodates joint effects). These findings highlight that part of the atrophy pattern in DLB is likely governed by polygenic interactions rather than isolated genetic effects. Nevertheless, the relatively small effect sizes overall are only slightly improved by the combination of different genes. This indicates that regional vulnerability to atrophy in DLB is probably determined by a variety of factors beyond gene expression, including connectivity and cell composition^50^. In fact, in a previous study by Rahayel and colleagues, only the combination of gene expression and structural connectivity predicted the atrophy pattern in idiopathic REM sleep behaviour disorder^20^. In DLB, our current study demonstrates that gene expression by itself was able to predict the atrophy pattern, possibly due to an increased atrophy in DLB as compared with idiopathic REM sleep behaviour disorder. Additionally, previous studies showed that cell composition contributes to a brain region’s vulnerability to alpha-synuclein, with oligodendrocytes and endothelial cells being protective against the atrophy in synucleinopathies^19,51^.

To test whether our results were specific to DLB or may be shared with those in other related diseases such as Alzheimer´s disease, we compared patients with and without A/T co-pathology as established biomarkers of Alzheimer´s disease. We found that, in DLB patients without A/T co-pathologies, regional vulnerability was mainly linked to *PARK7*, highlighting the role of alpha-synuclein in grey matter loss. However, in patients with A/T co-pathology, genes related to the accumulation of beta-amyloid and tau pathology, i.e., *MAPT* and *PSEN1,* also became important. This shows that the transcriptomic drivers of atrophy vary depending on which pathologies are present. These results suggest our findings are partly specific to alpha-synuclein as the main protein involved in DLB, but some atrophy findings may be related or accentuated by A/T co-pathology.

Some limitations should be considered. Firstly, in this study, we combined grey matter data of DLB patients from four different centres, allowing us to assemble one of the largest imaging data sets for DLB at the time. Since data from healthy controls was only acquired at the Mayo Clinic, we refrained from harmonizing the imaging data across centres to avoid introducing artificial differences. Instead, we tested the robustness of our results by conducting additional analyses, including only patients from the Mayo Clinic to prevent centre-related effects. Secondly, to limit the number of tests, we opted for a hypothesis-driven approach and exclusively included genes that were often associated with the accumulation of the common alpha-synuclein, beta-amyloid, and tau pathologies found in DLB patients. Nevertheless, we cannot dismiss the possibility that we did not include other genes contributing to the vulnerability of brain regions. As such, the expression of *LAG3*, *RAB5A*, and *LRRK2* was shown to mediate the synaptic transfer of alpha-synuclein and predicted atrophy in PD patients^17^. Further, expression of genes related to signalling pathways, metabolic function, and the immune system has been associated with DLB and its prodromal stages^52–55^. However, since the expression of the majority of the twelve included genes was correlated, we assume that additional genes would have exhibited a similar expression pattern, meaning they would not have significantly improved the model’s performance. Thirdly, we based gene expression patterns on microarray data from the Allen Human Brain Atlas, which were collected in healthy adults. Expression of the twelve genes included in this study might differ between DLB patients and healthy controls, due to different epigenetics, genetic variations, and cell type compositions^19,56,57^. In fact, Pietrzak and colleagues found 490 differentially expressed genes in an autopsy sample of DLB patients compared with healthy controls^57^. However, it remains unclear whether gene expression was consistently altered in specific brain regions or across the entire brain since the latter study obtained samples exclusively from the cingulate cortex. Apart from genes being differentially expressed, DLB patients may also carry different variants of genes, whose expression may increase vulnerability to the pathologies or may be protective^58–62^. Due to the lack of equivalent gene expression data derived from DLB patients, data extracted from the Allen Human Brain Atlas remains the best approximation of regional gene expression in DLB. Fourthly, we averaged normative gene expression values and grey matter volumes from the large number of included DLB patients (*n* = 164) and analysed their relationship across brain regions. This approach was chosen to reflect an average atrophy pattern typically observed in DLB patients. Importantly, this approach does not account for interindividual differences between DLB patients in atrophy or gene expression patterns. Hence, future studies should strive to extract gene expression and grey matter volumes in the same patients to investigate their relationship on the individual level. Fifthly, as shown in Fig. 3, differences in gene expression between cerebellar and subcortical regions on the one hand and cortical regions on the other may have acted as potential drivers of the regression results. We purposely included cerebellar and subcortical regions to capture the full spectrum of gene expression and atrophy patterns and highlight the importance of considering early sites affected by the alpha-synuclein pathology. Lastly, gene expression estimates derived from the Allen Human Brain Atlas rely on spatial interpolation, which may be less accurate for small subcortical structures where gene expression patterns may significantly differ across adjacent nuclei. However, this limitation is difficult to overcome with the currently available datasets.

In summary, our study provides the first evidence that normative regional expression of genes related to the accumulation of pathologies contributes to the atrophy in DLB. Apart from genes related to the underlying Lewy body pathology in DLB, our results highlight the importance of genes related to beta-amyloid and tau. This finding emphasises the relevance of co-pathologies in predicting atrophy progression and identifying potential targets for future gene therapies and disease-modifying treatments in DLB^63^.

## Methods

### Participants

This cross-sectional multicentre study included 164 DLB patients from Prague, Strasbourg, and Amsterdam centres within the European DLB (E-DLB) consortium and the Mayo Clinic DLB cohort. All included patients received a diagnosis of probable DLB based on the 2005 International Consensus Criteria, using interviews and available rating scales in each centre^64^. Patients were further characterised by the presence or absence of core clinical features of DLB, including idiopathic REM sleep behaviour disorder, Parkinsonism, visual hallucinations, and cognitive fluctuations. Additionally, global cognition was assessed with the MMSE. A/T co-pathologies were measured through cerebrospinal fluid in the three E-DLB centres and with positron emission tomography at the Mayo Clinic. Using centre-specific cut-points, positivity in beta-amyloid and/or tau biomarkers was interpreted as the presence of A/T co-pathologies. Patients who presented with acute delirium, terminal illness, prior stroke, psychotic or bipolar disorders, craniocerebral trauma and/or a recent diagnosis of a major somatic illness were excluded from the study. To delineate regional volumetric differences, we included 164 age- and sex-matched cognitively unimpaired controls from the Mayo Clinic Study of Aging.

### MRI acquisition

A high-resolution 3D T1-weighted magnetization-prepared rapid gradient (MPRAGE) echo sequence was acquired from all participants. At the Day Hospital of Geriatrics, Memory Resource and Research Centre (CMRR, Strasbourg, France), the Mayo Clinic (Rochester, US), and the Amsterdam University Medical Center (formerly known as VUmc, Amsterdam, Netherlands), images were acquired at a magnetic field strength of 3 T, whereas the Motol University Hospital (Prague, Czech Republic) used a 1.5 T scanner. A more detailed description of scanning parameters for each of the four centres is available in the supplementary information. (Table S1)

### MRI processing

To account for inter-site variability in scanner types and acquisition protocols, all MRI data were processed at the Mayo Clinic using previously established methods^65^. In short, the Mayo Clinic Adult Lifespan Template (MCALT; https://www.nitrc.org/projects/mcalt/) atlas was aligned with participants’ native MPRAGE space using Advanced Normalization Tools (ANTs). T1-MPRAGE images were then segmented into tissue classes using the unified segmentation algorithm in SPM12 (Wellcome Trust Center for Neuroimaging, London, UK), executed in Matlab (Mathworks, Natick, MA), with MCALT priors and settings. Following MCALT parcellation, volumes for 58 grey matter regions of interest (ROIs, including 41 cortical, 6 subcortical, 9 cerebellar, and 2 brainstem ROIs) were obtained for each participant by summing all grey matter probabilities from the segmentation within each region derived from the parcellation. We focused on left-hemispheric and midline brainstem regions since gene expression data from the Allen Human Brain Atlas^66^ was only available from left-hemispheric brain regions in the majority (4 out of 6) of donors. Additionally, total intracranial volume (TIV) was calculated as the sum of tissue probabilities of grey matter, white matter, and cerebrospinal fluid segmentations.

### Regional volume w-scores

To quantify regional volumetric differences between DLB patients and healthy controls, we calculated *w*-scores for each ROI, thus regressing out the partial effects of TIV, age, and sex found in matched healthy controls^67^:

1 $${w-{score}}_{{DLB\; patient}}=\frac{{{raw\; value}}_{{DLB\; patient}}-{{expected\; value}}_{{healthy\; control}}\left({for\; patien}{t}^{{\prime} }{s\; TIV},{age},{sex}\right)}{{{SD\; of\; residuals}}_{{healthy\; controls}}}$$

Regional *w*-scores were then averaged across all participants to identify a DLB-specific pattern of grey matter volume reductions.

### Regional gene expression

Regional microarray expression data were obtained from 6 post-mortem brains (1 female, 24-57 years) provided by the Allen Human Brain Atlas^66^, in line with common procedure in epidemic modelling and imaging transcriptomics studies^19,20,68,69^. Data were processed with the abagen toolbox (version 0.1.4^70^). First, microarray probes were reannotated using data provided by Arnatkevic̆iūtė and colleagues^71^. Next, probes were filtered based on their expression intensity relative to background noise, such that probes with intensity less than the background in ≥50% of samples across donors were discarded. When multiple probes indexed the expression of the same gene, we selected and used the probe with the most consistent pattern of regional variation across donors. The MNI coordinates of tissue samples were updated to those generated via non-linear registration using ANTs. Samples were assigned to the 58 MCALT brain regions if their MNI coordinates were within 2 mm of a given parcel. If a brain region was not assigned a tissue sample based on the above procedure, expression values were interpolated from the nearest tissue samples in surrounding regions. Inter-subject variation was addressed by normalizing tissue sample expression values across genes using a robust sigmoid function. Gene expression values were then normalized across tissue samples using an identical procedure. Samples assigned to the same brain region were averaged separately for each donor and then across donors.

For our imaging transcriptomics analyses, we selected normative expression profiles of ten risk genes identified by genome-wide association studies in DLB, Parkinson’s disease, and Alzheimer’s disease^25,29,45–47,72,73^. We focused on genes involved in the synthesis and degradation of alpha-synuclein as well as beta-amyloid and tau pathologies, specifically *APOE*, *APP*, *BIN1*, *GBA*, *LRP1*, *MAPT*, *PSEN1*, *PSEN2*, *SNCA*, and *TMEM175*. Additionally, we included *PARK7* and *PINK1*, due to their importance in clearance processes of alpha-synuclein^26,27^, providing a final number of 12 genes for statistical analysis.

### Statistical analyses

All statistical analyses were performed in Matlab 2019b, except for random forest regression, which was conducted in R version 4.3.0 using the *randomForest* package. Differences in demographic and clinical variables between DLB patients and healthy controls were tested using two-tailed *t*-tests and χ^2^-tests for continuous and categorical variables, respectively. An α-level of *p* < 0.05 (two-tailed) denoted statistical significance.

To explore whether observed negative w-scores in DLB patients would reflect atrophy in DLB patients compared to healthy controls, we compared regional volumes of all 58 brain regions using t-tests. Additionally, we conducted pairwise Pearson’s correlations on gene expression values to explore the interplay between the twelve genes. For the first aim, we assessed the individual association of normative regional gene expression with regional grey matter volumes in DLB, using separate linear regression models for each of the twelve genes. To account for spatial autocorrelation in gene expression data, we generated 10’000 spatially constrained surrogate maps per gene using *BrainSMASH* in Python 3.12, preserving the spatial autocorrelation structure of the empirical gene expression data. Gene–atrophy associations were tested using linear regression, and *p*-values were computed relative to null distributions derived from surrogate maps. The Benjamin-Hochberg method was applied to control the false discovery rate across the 12 genes.

For our second aim, we used random forest regression to evaluate the combined prediction of normative expression of the twelve genes on regional grey matter volume in DLB. Regional volumetric *w*-scores served as the outcome variable while normative regional expressions of the twelve genes were entered as predictors in the random forest regression. We used the default value for the number of predictors randomly sampled at each split for random forest regression (*mtry*), number of predictors/3 = 4, as tuning the value did not significantly improve model performance. The number of trees (*ntree*) was set to 500 as the error did not significantly decrease with more trees. The minimal number of observations in each terminal node (*nodesize*) was set to 2. The performance of the model is reported as the out-of-bag mean squared error (OOB MSE) and the percentage of variance in the response variable that was explained by the model (% Var(y)). Additionally, we report the importance of predictors in the prediction of the outcome variable in terms of increase in mean squared error. To account for spatial autocorrelation in the random forest regression analyses, we randomly selected 1’000 of the previously created surrogate maps. Statistical significance for global model performance and individual variable importance was determined by comparing the empirical values against null distributions derived from the surrogates.

Due to the multi-centre nature of our cohort, we tested the robustness of our results by repeating all analyses in several subsamples of our cohort. To preclude that centre-specific effects were driving the results, we repeated the analyses, including only patients and controls from the Mayo Clinic cohort, which was the biggest sub-cohort in this study.

To test whether our results were specific to DLB or driven by common co-pathologies typically associated with Alzheimer’s disease, we repeated the analyses in subgroups of DLB patients with and without A/T co-pathologies, including patients from all centres.

### Ethics declaration

Local ethics committees at each participating E-DLB centre and the Mayo Clinic Institutional Review Board approved the study. Additionally, the Swedish Ethical Review Authority approved data management and analyses (2022-01251-02). In compliance with the Declaration of Helsinki, all participants or appropriate surrogates provided written informed consent prior to participation in the study.

## Supplementary information

Supplementary material

## Data Availability

The MRI data that support the findings of this study are available through the E-DLB consortium (https://www.e-dlb.com) and the Mayo Clinic (https://www.mayo.edu/research/labs/aging-dementia-imaging/overview) for qualified researchers upon request. The included gene expression data is publicly available from the Allen Human Brain Atlas (https://human.brain-map.org/).

## Code availability

The underlying code for this study is not publicly available but may be made available to qualified researchers upon request to the corresponding author.
