# Supplementary material for: Regional gene expression and brain atrophy in dementia with Lewy bodies: an imaging transcriptomics study
Source: NPJ Parkinsons Dis. 2026 Apr 16;12:96. doi: 10.1038/s41531-026-01355-2 (PMC13087014; doi:10.1038/s41531-026-01355-2)
Supplement: Supplementary file 1 — Supplementary material [file 41531_2026_1355_MOESM1_ESM.pdf]

Supplementary material

			European DLB Consortium	
	**Mayo Clinic (n = 67)**	**VUmc, Amsterdam (n = 34)**	**CMRR, Strasbourg (n = 34)**	**Motol University Hospital, Prague (n = 29)**
Vendor	General Electric	General Electric	Siemens	Siemens
Scanner model	Discovery 750, Signa	Discovery 750, Signa	Verio	Avanto
Field strength, T	3	3	3	1.5
Type	3D	3D	3D	3D
TR, ms.	2300	8	1900	2000
TE, ms.	3	3	2.53	3.08
TI, ms.	900	450	900	1100
FA, °	8	12	9	15
Voxel size, mm	1 x 1 x 1.2	0.98 x 0.98 x 1	1 x 1 x 1	0.98 x 0.98 x 1

Table S1 MRI scanner parameters for T1 sequences.

**Brain regions**	**All**	**A/T-**	**A/T+**	**Mayo**
Amygdala	**-0.995**	**-0.810**	**-1.133**	**-0.847**
Angular	**-0.879**	**-0.886**	**-1.038**	**-0.675**
Calcarine	**-0.587**	**-0.769**	**-0.700**	-0.193
Caudate	**-0.645**	**-0.740**	**-0.770**	-0.199
Cerebellum Lobule X	**-0.398**	**-0.470**	**-0.416**	-0.080
Cerebellum Lobule III	**-0.304**	**-0.485**	-0.202	-0.075
Cerebellum Lobules IV-V	-0.101	-0.199	0.011	-0.032
Cerebellum Lobule VI	**-0.347**	**-0.437**	-0.414	-0.117
Cerebellum Lobule VIIb	**-0.522**	**-0.626**	**-0.599**	-0.312
Cerebellum Lobule VIII	-0.364	**-0.581**	-0.325	-0.056
Cerebellum Lobule IX	**-0.388**	**-0.488**	-0.329	-0.276
Cerebellum Crus I	**-0.385**	**-0.531**	-0.323	-0.198
Cerebellum Crus II	**-0.546**	**-0.668**	-0.472	-0.541
Cingulum_Ant	-0.358	-0.287	-0.296	-0.491
Cingulum_Mid	-0.323	-0.148	-0.502	-0.252
Cingulum_Post	**-0.622**	**-0.688**	**-0.614**	**-0.515**
Cuneus	**-0.647**	**-0.739**	**-0.702**	-0.307
Dorsal_Mesopontine	-0.234	-0.012	-0.168	**-0.553**
Entorhinal_Cortex	**-0.415**	-0.309	-0.451	-0.256
Frontal_Inf_Oper	**-0.352**	-0.218	**-0.483**	**-0.488**
Frontal_Inf_Orb	**-0.579**	**-0.522**	-0.424	**-0.611**
Frontal_Inf_Tri	**-0.439**	**-0.377**	**-0.479**	-0.400
Frontal_Med_Orb	**-0.650**	-0.452	**-0.834**	-0.453
Frontal_Mid	**-0.843**	**-0.851**	**-0.918**	**-0.761**
Frontal_Mid_Orb	**-0.609**	**-0.419**	**-0.631**	**-0.573**
Frontal_Sup	**-0.805**	**-0.691**	**-0.910**	**-0.759**
Frontal_Sup_Medial	**-0.850**	**-0.771**	**-0.968**	-0.550
Frontal_Sup_Orb	**-0.420**	**-0.440**	-0.330	-0.407
Fusiform	**-1.016**	**-0.829**	**-1.144**	**-0.983**
Heschl	**-0.435**	**-0.457**	-0.366	-0.324
Hippocampus	**-0.550**	**-0.544**	-0.473	-0.345
Insula	**-0.867**	**-0.958**	**-0.845**	-0.613
Lingual	**-0.875**	**-1.017**	**-1.041**	-0.289
Occipital_Inf	**-0.617**	**-0.609**	**-0.645**	-0.431
Occipital_Mid	**-1.082**	**-1.033**	**-1.126**	**-1.063**
Occipital_Sup	**-0.637**	**-0.673**	**-0.576**	**-0.571**
Olfactory	-0.239	-0.188	-0.175	-0.467
Pallidum	0.030	0.041	-0.122	0.042
Paracentral_Lobule	-0.173	-0.185	-0.017	-0.180
ParaHippocampal	**-0.499**	**-0.427**	-0.448	-0.425
Parietal_Inf	**-0.655**	**-0.541**	**-0.690**	**-0.734**
Parietal_Sup	**-0.826**	**-0.771**	**-0.795**	**-0.911**
Pons	**-0.469**	**-0.437**	**-0.474**	**-0.461**
Postcentral	-0.380	**-0.577**	-0.093	-0.513
Precentral	-0.334	-0.277	-0.209	-0.525
Precuneus	**-0.955**	**-0.857**	**-0.994**	**-0.971**
Putamen	**-0.469**	**-0.556**	**-0.598**	-0.038
Rectus	**-0.652**	**-0.719**	-0.499	**-0.639**
Retrosplenial	**-0.510**	**-0.499**	**-0.553**	-0.360
Rolandic_Oper	**-0.493**	**-0.469**	-0.456	-0.531
Supp_Motor_Area	-0.278	-0.229	-0.245	-0.304
SupraMarginal	**-0.646**	**-0.439**	**-0.704**	**-0.732**
Temporal_Inf	**-0.922**	**-0.877**	**-0.934**	**-0.847**
Temporal_Mid	**-0.594**	**-0.608**	-0.440	**-0.774**
Temporal_Pole_Mid	**-0.423**	**-0.374**	-0.373	**-0.418**
Temporal_Pole_Sup	-0.259	-0.194	-0.243	-0.244
Temporal_Sup	**-0.480**	**-0.478**	-0.485	-0.538
Thalamus	**-0.524**	**-0.434**	**-0.542**	**-0.696**

Table S2 Average atrophy (*w*-scores) in left hemisphere of all DLB patients (*n*=164), DLB patients without A/T co-pathologies (A/T-, *n*=56), DLB patients with A/T co-pathologies (A/T+, *n*=66), and DLB patients from the Mayo Clinic (Mayo, *n*=67) compared to healthy controls. Numbers in bold indicate significant differences between DLB patients and healthy controls based on *t*-tests.

**Brain regions**	*APOE*	*APP*	*BIN1*	*GBA*	*LRP1*	*MAPT*	*PARK7*	*PINK1*	*PSEN1*	*PSEN2*	*SNCA*	*TMEM 175*
Amygdala	0.856	0.623	0.496	0.369	0.711	0.270	0.412	0.378	0.486	0.555	0.424	0.700
Angular	0.357	0.501	0.395	0.479	0.334	0.721	0.574	0.674	0.423	0.470	0.700	0.557
Calcarine	0.263	0.530	0.461	0.425	0.422	0.581	0.596	0.574	0.435	0.351	0.507	0.492
Caudate	0.906	0.494	0.478	0.404	0.566	0.372	0.550	0.369	0.540	0.426	0.214	0.592
Cerebellum Lobule X	0.486	0.046	0.198	0.132	0.428	0.256	0.423	0.122	0.238	0.522	0.443	0.242
Cerebellum Lobule III	0.647	0.140	0.231	0.148	0.541	0.249	0.547	0.238	0.427	0.628	0.320	0.380
Cerebellum Lobules IV-V	0.625	0.056	0.145	0.093	0.530	0.369	0.547	0.137	0.321	0.541	0.351	0.301
Cerebellum Lobule VI	0.547	0.057	0.146	0.075	0.537	0.310	0.479	0.127	0.274	0.559	0.381	0.265
Cerebellum Lobule VIIb	0.526	0.010	0.210	0.111	0.597	0.370	0.487	0.091	0.228	0.597	0.500	0.289
Cerebellum Lobule VIII	0.550	0.066	0.235	0.121	0.589	0.345	0.444	0.107	0.259	0.615	0.459	0.295
Cerebellum Lobule IX	0.469	0.024	0.230	0.081	0.535	0.318	0.336	0.080	0.302	0.460	0.479	0.221
Cerebellum Crus I	0.575	0.025	0.119	0.075	0.507	0.376	0.455	0.104	0.224	0.597	0.447	0.281
Cerebellum Crus II	0.592	0.020	0.176	0.085	0.584	0.344	0.418	0.095	0.254	0.534	0.476	0.265
Cingulum_Ant	0.652	0.568	0.529	0.654	0.459	0.675	0.599	0.657	0.455	0.466	0.801	0.530
Cingulum_Mid	0.404	0.515	0.529	0.625	0.295	0.739	0.612	0.671	0.462	0.573	0.808	0.576
Cingulum_Post	0.411	0.522	0.484	0.553	0.378	0.715	0.560	0.650	0.369	0.459	0.675	0.546
Cuneus	0.328	0.595	0.408	0.419	0.444	0.602	0.532	0.603	0.483	0.362	0.495	0.490
Dorsal_Mesopontine	0.788	0.734	0.579	0.455	0.796	0.184	0.254	0.397	0.773	0.758	0.299	0.793
Entorhinal_Cortex	0.800	0.534	0.561	0.614	0.536	0.757	0.647	0.682	0.513	0.445	0.697	0.458
Frontal_Inf_Oper	0.390	0.515	0.544	0.642	0.331	0.639	0.647	0.741	0.434	0.482	0.796	0.564
Frontal_Inf_Orb	0.397	0.581	0.532	0.613	0.338	0.651	0.647	0.717	0.410	0.450	0.785	0.577
Frontal_Inf_Tri	0.366	0.440	0.634	0.644	0.244	0.592	0.610	0.752	0.461	0.397	0.777	0.551
Frontal_Med_Orb	0.634	0.515	0.546	0.626	0.346	0.742	0.673	0.688	0.422	0.492	0.861	0.423
Frontal_Mid	0.481	0.491	0.527	0.647	0.320	0.658	0.648	0.763	0.424	0.458	0.752	0.506
Frontal_Mid_Orb	0.429	0.511	0.599	0.597	0.313	0.576	0.634	0.646	0.521	0.354	0.790	0.542
Frontal_Sup	0.494	0.568	0.457	0.636	0.346	0.688	0.566	0.700	0.448	0.505	0.739	0.477
Frontal_Sup_Medial	0.488	0.549	0.591	0.659	0.311	0.707	0.530	0.659	0.517	0.419	0.764	0.482
Frontal_Sup_Orb	0.531	0.525	0.570	0.652	0.266	0.633	0.611	0.719	0.399	0.410	0.844	0.447
Fusiform	0.544	0.524	0.563	0.648	0.410	0.710	0.577	0.678	0.462	0.467	0.828	0.525
Heschl	0.370	0.547	0.571	0.501	0.371	0.638	0.598	0.631	0.522	0.392	0.711	0.504
Hippocampus	0.534	0.742	0.578	0.476	0.735	0.324	0.284	0.364	0.453	0.624	0.432	0.641
Insula	0.533	0.555	0.594	0.666	0.446	0.751	0.598	0.691	0.487	0.532	0.859	0.503
Lingual	0.275	0.537	0.484	0.410	0.428	0.652	0.572	0.616	0.469	0.369	0.555	0.550
Occipital_Inf	0.348	0.513	0.518	0.441	0.344	0.695	0.574	0.629	0.418	0.352	0.576	0.513
Occipital_Mid	0.400	0.561	0.476	0.492	0.379	0.679	0.580	0.657	0.428	0.340	0.640	0.480
Occipital_Sup	0.327	0.587	0.470	0.453	0.399	0.648	0.564	0.578	0.513	0.328	0.534	0.473
Olfactory	0.539	0.534	0.512	0.687	0.312	0.703	0.639	0.677	0.325	0.469	0.887	0.583
Pallidum	0.924	0.558	0.600	0.447	0.607	0.375	0.429	0.304	0.734	0.623	0.245	0.537
Paracentral_Lobule	0.324	0.505	0.484	0.575	0.311	0.647	0.621	0.733	0.408	0.530	0.726	0.597
ParaHippocampal	0.613	0.595	0.562	0.625	0.467	0.602	0.508	0.621	0.487	0.383	0.800	0.481
Parietal_Inf	0.446	0.449	0.518	0.621	0.379	0.777	0.611	0.736	0.468	0.426	0.712	0.463
Parietal_Sup	0.415	0.527	0.413	0.544	0.363	0.690	0.615	0.707	0.424	0.432	0.669	0.456
Pons	0.608	0.623	0.571	0.383	0.738	0.166	0.300	0.407	0.771	0.777	0.281	0.736
Postcentral	0.334	0.445	0.482	0.530	0.366	0.661	0.653	0.726	0.489	0.416	0.641	0.520
Precentral	0.376	0.476	0.438	0.615	0.372	0.667	0.613	0.756	0.489	0.523	0.660	0.562
Precuneus	0.434	0.473	0.489	0.547	0.361	0.713	0.531	0.659	0.412	0.418	0.680	0.463
Putamen	0.788	0.628	0.561	0.405	0.612	0.393	0.301	0.336	0.681	0.482	0.339	0.575
Rectus	0.517	0.614	0.580	0.661	0.388	0.613	0.643	0.682	0.466	0.434	0.840	0.550
Retrosplenial	0.413	0.494	0.422	0.526	0.329	0.695	0.571	0.560	0.351	0.476	0.642	0.436
Rolandic_Oper	0.398	0.586	0.426	0.617	0.362	0.706	0.647	0.683	0.411	0.494	0.776	0.525
Supp_Motor_Area	0.453	0.549	0.426	0.660	0.381	0.670	0.612	0.743	0.435	0.567	0.779	0.540
SupraMarginal	0.398	0.499	0.472	0.580	0.370	0.729	0.610	0.697	0.464	0.420	0.749	0.499
Temporal_Inf	0.583	0.501	0.531	0.624	0.360	0.750	0.597	0.687	0.466	0.442	0.821	0.492
Temporal_Mid	0.490	0.514	0.578	0.574	0.339	0.740	0.572	0.648	0.495	0.461	0.774	0.523
Temporal_Pole_Mid	0.737	0.505	0.533	0.711	0.312	0.710	0.665	0.728	0.394	0.476	0.874	0.453
Temporal_Pole_Sup	0.664	0.529	0.572	0.681	0.395	0.657	0.587	0.665	0.444	0.491	0.870	0.479
Temporal_Sup	0.457	0.508	0.515	0.565	0.345	0.702	0.637	0.660	0.469	0.449	0.790	0.482
Thalamus	0.801	0.554	0.667	0.493	0.642	0.317	0.372	0.380	0.693	0.777	0.168	0.618

Table S3 Average regional gene expression pattern of twelve included genes derived from healthy controls in Allen Human Brain Atlas.

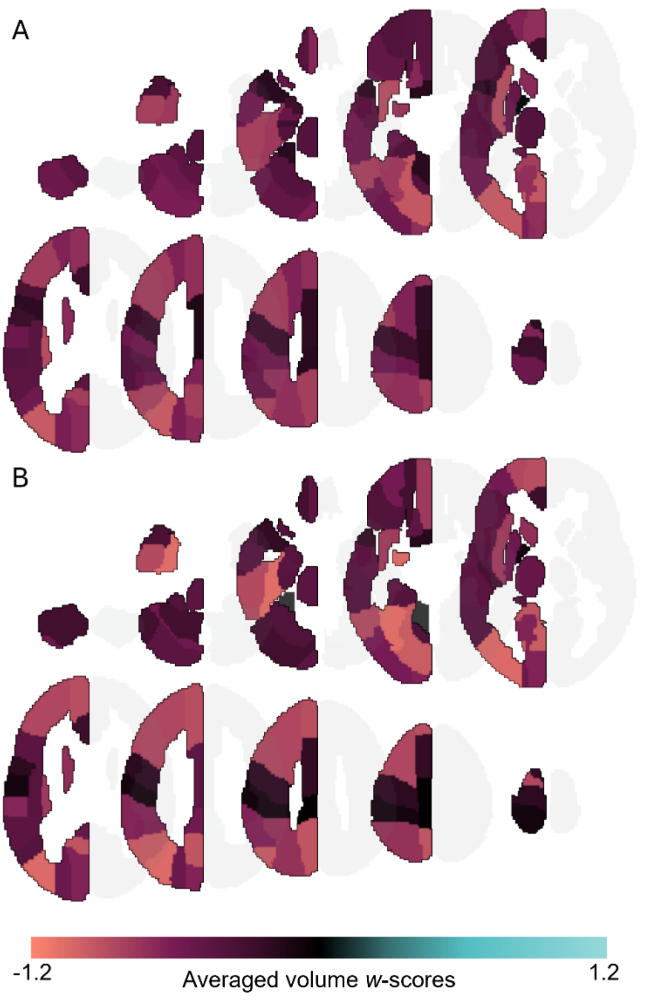

Figure S1 Average atrophy pattern (*w*-scores) in left hemisphere of **A** DLB patients without A/T co-pathologies (*n*=56) and **B** DLB patients with A/T co-pathologies (*n*=66) compared to healthy controls.

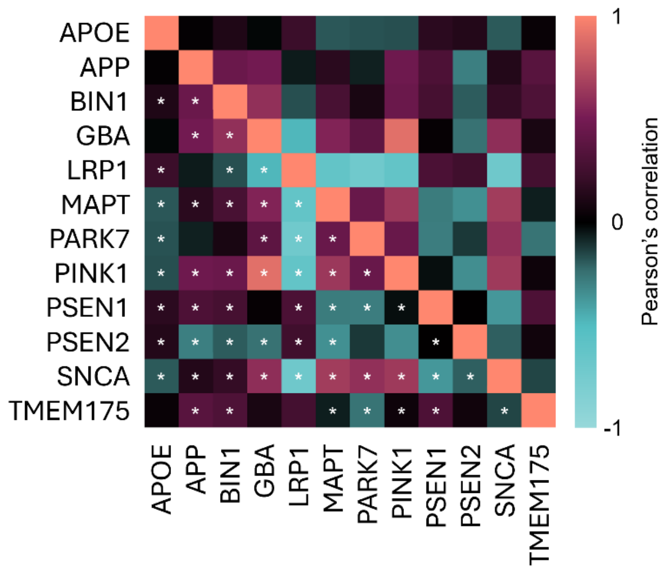

Figure S2 Correlations between normative expressions of twelve genes across 58 brain regions. Squares with asterisks indicate significant correlations.

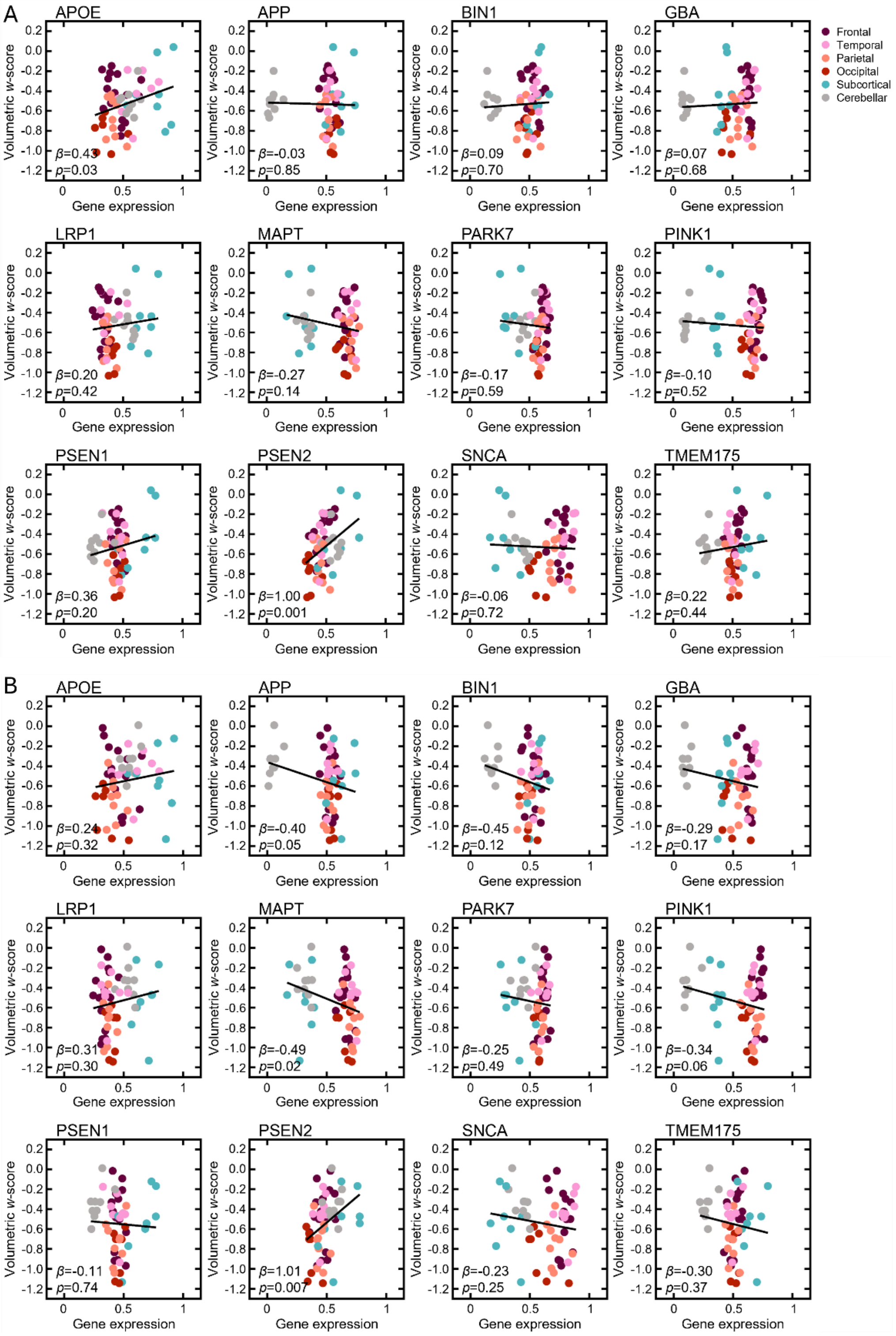

Figure S3 Simple linear regression of gene expression on atrophy (*w*-scores) across 58 brain regions in **A** DLB patients without A/T co-pathologies (n=56) and **B** DLB patients with A/T co-pathologies (n=66). Colours denote regions' affiliation with lobes and major brain structures. Panels include beta coefficients β and p-values adjusted for spatial autocorrelation $p_{spatial}$.

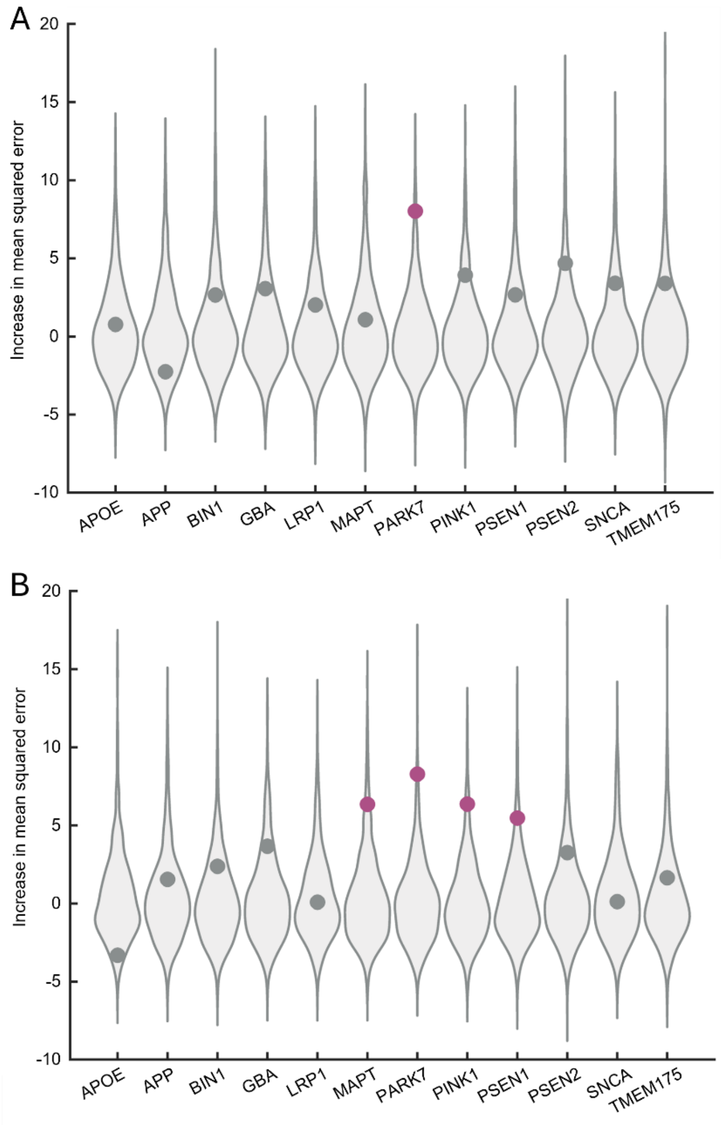

Figure S4 Importance of normative gene expressions as predictors of atrophy in random forest regression analysis across 58 brain regions in **A** DLB patients without A/T co-pathologies (56) and **B** DLB patients with A/T co-pathologies (*n*=66). Circular markers represent empirical importance of each gene with plum markers indicating significance $p_{spatial}<0.05$ relative to null distribution. Grey violins represent null distributions of variable importance across 1'000 surrogate maps.
